# Skeletal stability after 2-jaw surgery via surgery-first approach in facial asymmetry patients using CBCT

**DOI:** 10.1186/s40902-020-00253-8

**Published:** 2020-04-09

**Authors:** Dae Seok Hwang, Jeong Seok Seo, Hong Seok Choi

**Affiliations:** 1 Dental Research Institute, Department of Oral and Maxillofacial Surgery, School of Dentistry, Pusan National University, Pusan National University Dental Hospital, Dental Research Institute, Beomeori, Mulgeum, Yangsan, Kyoungsangnamdo 50612 South Korea; 2 Department of Dental Clinic, Bongseng Hospital, 401, Jungang-daero, Dong-gu, Busan South Korea; 3 Department of Dental Clinic, Kosin University Hospital, 262, Gamcheon-ro, Seo-gu, Busan 49267 South Korea

**Keywords:** Facial asymmetry, Orthognathic surgery, Surgery-first approach, Skeletal stability, 3-Dimensional analysis

## Abstract

**Background:**

The purpose of this study is to compare the skeletal stability of two-jaw surgery via surgery-first approach with conventional two-jaw surgery in facial asymmetry patients by measuring the skeletal changes after surgery from a three-dimensional analysis. From January 2010 to January 2014, 40 patients with facial asymmetry who underwent two-jaw surgery in Pusan National University Hospital were included in this study. They were classified into experimental group (*n* = 20) who underwent two-jaw surgery via surgery-first approach and control group (*n* = 20) who underwent conventional two-jaw surgery. After selection of 24 landmarks and the construction of horizontal and sagittal, coronal reference planes, changes in 10 linear measurements and 2 angular measurements were compared between the surgery-first approach and conventional groups in the preoperative, immediate postoperative, and postoperative periods. The paired *t* test and Student *t* test were used for statistical analysis. The mean and standard deviation of the measurement were calculated for the experimental and control groups.

**Results:**

The statistical analysis showed that changes in skeletal measurements were similar between the surgery-first approach and conventional groups, according to each period. However, U1-SRP measurement showed statistically significant changes in surgery-first approach groups at postsurgical change (T1 to T2). Also, the mean treatment duration in the treatment group was 15.9 ± 5.48 months whereas that in the control group was 32.9 ± 14.05 months.

**Conclusion:**

In facial asymmetry patients, similar results were observed in the postoperative skeletal stability when 2-jaw surgery via surgery-first approach was compared with conventional 2-jaw surgery. However, significant lateral deviation of upper incisor midline was observed. In addition, a shorter average treatment duration was observed. To stabilize the unstable occlusion after surgery, increased wearing of the stent and proactive rubber guidance will be needed.

## Introduction

Facial symmetry is consistent with the sizes and shapes of the left and right sides of the face [1]. It is a necessary element of the human face, and asymmetric faces are generally considered non-appealing and dissonant [2]. As first observed by a Greek artist, facial asymmetry can have negative effects on facial harmony and beauty [3]. In previous studies, among patients with dentofacial deformity, 34% were reported to have had facial asymmetry in the USA [4], and 25% in Hong Kong [5], and especially in groups with skeletal class III, 42.3% in South Korea [6] and 40% in the USA [4, 7]. The number of facial asymmetric patients is increasing, and the degree of asymmetry becomes more complex with time. Therefore, a more accurate diagnosis and treatment planning are needed [8].

In most cases, the existence and degree of facial asymmetry can be diagnosed based on a frontal cephalogram [9]. Displacement of the mentum is a common form of facial asymmetry that occurs due to the difference between the lengths of the left and right mandibular bodies. Identifying such structural issues is important in establishing a treatment plan, but frontal cephalograms do not provide sufficient information for identifying the source of asymmetry or determining an appropriate treatment plan. Two-dimensional analysis has limitations: a 3D structure is projected on a 2D cross-section, which may cause image distortion and an error in the enlargement ratio [10]. The development of 3D computed tomography has significantly reduced such errors and has facilitated a 3D understanding of the structure [11].

Orthognathic surgery traditionally consists of preoperative orthodontic treatment, orthognathic surgery, and postoperative orthodontic treatment. The need for preoperative orthodontic treatment is based on the fact that the position of the jaws can be limited by the inadequate tooth arrangement at the time of surgery. A disadvantage of preoperative orthodontic treatment is its long duration of 7–47 months, which increases the chances of developing dental caries, gingival recession, and root resorption [12, 13]. In 1991, Brachvogel et al. [14] proposed a surgery-first approach to overcome such disadvantage. He argued that the surrounding normalized soft tissues after the surgery in such approach facilitate the orthodontic movement of the teeth and reduce the overall treatment duration. In 2010, Liao et al. [15] reported no difference in stability when the surgery-first approach was implemented in skeletal class III and open-bite patients. In 2010, however, Baek et al. [16] reported that the successful implementation of the surgery-first approach requires extensive clinical experience for the accurate assessment of skeletal disharmony and the correct prediction of the treatment outcome, and that the instability of postoperative occlusion leads to skeletal instability and difficulties in postoperative orthodontic treatment. In 2010, Choi et al. [17] reported that results similar to those of the traditional method can be obtained in a dental model of the surgery-first approach using preoperative simulation. In 2011, Ko et al. [18] reported no significant difference in skeletal correction, postoperative regression, and treatment duration when the surgery-first approach was implemented in class III malocclusion patients. In 2014, Hernández-Alfaro et al. [19] reported that the surgery-first approach significantly reduced the treatment duration and achieved a high level of patient satisfaction, but that it required careful patient selection, accurate treatment planning, and active interaction with the orthodontist. As such, various studies have reported contradictory results pertaining to orthognathic surgery through the surgery-first approach in class III malocclusion patients.

As opposed to traditional preoperative orthodontic treatment, it is easy to move the teeth by shortening the entire treatment period by performing the surgery first and by performing orthodontic treatment under improved postoperative musculoskeletal relationship. In addition to reducing the duration of orthodontic treatment, it is also caused by postoperative bone metabolism. Several studies have reported rapid bone response after surgery as a result of RAP (regional acceleratory phenomenon) [20, 21].

There are few reports related to the use of orthognathic surgery in facial asymmetry patients due to the difficulty of predicting the outcome of the treatment and of setting up the final occlusion of the maxillary and mandibular molars. In this study, orthognathic surgery was performed through the surgery-first approach. In addition, the skeletal stability values were compared by measuring the skeletal changes before and after surgery using the 3D computed tomography findings.

The purpose of this study is to compare skeletal stability of two-jaw surgery via surgery-first approach with conventional two-jaw surgery in facial asymmetry patients by measuring the skeletal changes after surgery from three-dimensional analysis.

## Patients and methods

### Patients

The study involved patients with at least 3-mm occlusal canting due to the preoperative difference between the vertical dimensions of the left and right maxilla among the patients who had been diagnosed with facial asymmetry at the Oral and Maxillofacial Surgery Department of Pusan National University Hospital between January 2010 and January 2014 and who had completed orthodontic treatment after undergoing orthognathic surgery. Occlusal canting criterion mentioned above was defined as the difference in the vertical distance from the mesiobuccal cusp of the each maxillary first molar to the horizontal reference plane. Of these, 20 subjects on whom the surgery-first approach was implemented were randomly placed in the treatment group whereas 20 subjects on whom the surgery-first approach was not implemented were randomly placed in the control group. The patients with maxillary premolar extraction, cleft lip and palate, and other craniofacial syndromes or TMJ disorders were excluded. All the patients underwent Le Fort I osteotomy, ascending mandibular sagittal split osteotomy, and orthognathic surgery using rigid fixation by the same surgeon. For the surgical occlusion, the anteroposterior position was set to the class I key. The increase in vertical height due to the premature interference of the second molar or premolar was also addressed in the operation. For this, the amount of forward movement of the mandibular first molar around the mandibular condyle was considered. Vertical overlying in the anterior was determined based on the facial form, the positions of the anterior maxilla, the curve of speed of the mandibular dentition, and the degree of crowding. After a 3-day postoperative intermaxillary fixing, mouth opening exercise was performed. A surgical stent was used for approximately 4–6 weeks and was adjusted every week considering the movement of the teeth.

### Methods

Using dental cone-beam CT (DCT pro, Vatech, Seoul, South Korea), the images of the facial form of the patients were obtained before surgery (T0), immediately after surgery (T1), and 6 months after surgery (T2). The DCT images obtained from the patients were converted to the DICOM format, were reconstructed into a 3D image using OnDemand (Cybermed, Seoul, South Korea), and were then measured. The horizontal and sagittal planes were used as references whereas the plane that was perpendicular to these two planes was used as the coronal reference (Tables 1 and 2). For the analysis of the skeletal stability, a total of 12 measurements were made, including the four categories of maxillary measurements (A-SRP, U1-SRP, R maxillary height, and L maxillary height) and the eight categories of mandibular measurements (Me-SRP, Pog-CP, R body height, L body height, R ramus length, L ramus length, R frontal ramal inclination, and L frontal ramal inclination) (Table 3).

**Table 1:** Reference planes

Reference planes	
HRP (horizontal reference plane)	The plane constructed by connecting both sides of Or and Po_Rt._
SRP (sagittal reference plane)	Perpendicular to HRP passing through Na and Ba
CP (coronal plane)	Perpendicular to both the HRP and SRP
MP (mandibular plane)	The plane constructed by Me and both sides of Ag

**Table 2:** Reference points

Reference points	
Na(Nasion)	Most anterior point of the frontonasal suture
U1	Contact point of maxillary central incisors
A	Most posterior point on curve between ANS and prosthion
Pog(Pogonion)	Most anterior midpoint of symphysis of mandible
Me(Menton)	Most inferior point on the symphysis outline
Ba(Basion)	Most anterior, inferior point of foramen magnum
Or_Rt._(Orbitale Rt.)	Most inferior point of the Rt. orbital rim
Or_Lt._(Orbitale Lt.)	Most inferior point of the Lt. orbital rim
Po_Rt._(Porion Rt.)	Most superior point of the Rt. external auditory meatus
Po_Lt._(Porion Lt.)	Most superior point of the Lt. external auditory meatus
Cd_supRt._(Condylion Superior Rt.)	Most superior point of Rt. condyle head
Cd_supLt._(Condylion Superior Lt.)	Most superior point of Lt. condyle head
Cd_latRt._(Condylion Lateral Rt.)	Most lateral point of Rt. condyle head
Cd_latLt._(Condylion Lateral Lt.)	Most lateral point of Lt. condyle head
Go_latRt._(Gonion Lateral Rt.)	Most lateral point of Rt. gonion area
Go_latLt._(Gonion Lateral Lt.)	Most lateral point of Lt. gonion area
Go_infRt._(Gonion Inferior Rt.)	Most inferior point of Rt. gonion area
Go_infLt._(Gonion Inferior Lt.)	Most inferior point of Lt. gonion area
Ag_Rt._(Antegonion Rt.)	Deepest point of Rt. antegonial notch of mandible
Ag_Lt._(Antegonion Lt.)	Deepest point of Lt. antegonial notch of mandible
U6CP_Rt_	The mesiobuccal cusp of the Rt. maxillary first molar
U6CP_Lt_	The mesiobuccal cusp of the Lt. maxillary first molar
L3CP_Rt_	The cuspal tip of the Rt. mandibular canine
L3CP_Lt._	The cuspal tip of the Lt. mandibular canine

**Table 3:** Measurements

Measurements	
A-SRP	Distance from A point to SRP (sagittal reference plane)
U1-SRP	Distance from U1 to SRP (sagittal reference plane)
Me-SRP	Distance from Me to SRP (sagittal reference plane)
Pog-CP	Distance from Pog to CP (coronal plane)
R body height	Distance from L3CP_Rt._ to MP (mandibular plane)
L body height	Distance from L3CP_Lt._ to MP (mandibular plane)
R maxillary height	Distance from U6CP_Rt._ to HRP (horizontal reference plane)
L maxillary height	Distance from U6CP_Lt._ to HRP (horizontal reference plane)
R ramus length	Distance from Cd_supRt._ to Go_infRt._
L ramus length	Distance from Cd_supLt._ to Go_infLt_
R frontal ramal inclination	Angle between Cd_latRt._-Go_latRt._ to SRP (sagittal reference plane)
L frontal ramal inclination	Angle between Cd_latLt._-Go_latLt._ to SRP (sagittal reference plane)

This study was a retrospective study using the patients’ medical records and radiographic materials with the approval of the Pusan National University Hospital Clinical Trial Review Committee (IRB No. PNUH-2015-017).

### Operation

One week before the orthognathic surgery, an orthodontic device was attached to start the orthodontic treatment and a model for the preparation of a stent for surgery was prepared. In surgery, after nasotracheal intubation, local anesthesia was performed with lidocaine containing 1:100,000 epinephrine in the mucobuccal fold of the maxilla and mandible. The procedure of Le Fort I osteotomy involved a general maxillary vestibular incision from the midline to the second molar, followed by tissue detachment and subperiosteal dissection to expose the orbital ridge downward and malar prominence while preserving the orbital nerve. After elevating the mucosal periosteum and nasal mucosa, the osteotomy line was created by using a pencil. Osteotomy was performed by using a reciprocating saw. The lateral aspect of the nasal wall and the nasal septum were fractured via the nasal osteotome. The pterygoid plate was separated from the palate by using a strong curved chisel. After confirming the movement of the maxilla, an intermediate wafer was used to move it to the planned position. The fixation of the maxilla was done by using four L-shaped fixation monocortical plates. One fixation is applied to the paranasal part, both left and right, and one fixation to the buttress part. The mucosa is sutured by using a 3-0 Vicryl suture and a 4-0 Ethilon suture. Maxillomandibular fixation (MMF) was performed for 5 days after surgery, and then lip movement and mouth opening were performed. The surgical stent was periodically adjusted by occlusal adjustment to promote teeth movement. After 6 weeks, the surgical stent was completely removed and an active orthodontic treatment was performed.

### Statistical analysis

The statistical significance was tested using a paired *t* test for the changes in the measurements over time in the treatment and control groups. The average difference between the changes in the measurements in the treatment and control groups over time was tested using Student’s *t* test. All the statistical analyses were performed using SPSS (SPSS, Chicago, IL, USA). A *P* value of less than 0.05 was deemed statistically significant.

## Results

The mean values and standard deviations were calculated for the measurements that changed over time in the treatment and control groups, and their significance values were compared. The treatment group consisted of 20 subjects (10 males, 10 females) with a mean age of 22.45 ± 0.92 years. The control group, on the other hand, consisted of 20 subjects (6 males, 14 females) with a mean age of 22.85 ± 0.98 years. The mean value of occlusal canting in the treatment group was 3.02 ± 0.17 mm whereas that in the control group was 4.15 ± 0.34 mm. The mean treatment duration in the treatment group was 15.9 ± 5.48 months whereas that in the control group was 32.9 ± 14.05 months (Table 4).

**Table 4:** Demographic data of the subjects

	Experimental group, mean (SD)	Control group, mean (SD)
Sample size (*n*)	20	20
Sex (male/female)	10/10	6/14
Age (years)	22.45 (0.92)	22.85 (0.98)
Amount of occlusal canting (mm)	3.02 (0.17)	4.15 (0.34)
Total treatment time (months)	15.9 (5.48)	32.9 (14.05)

The treatment group consisted of 20 subjects (10 males, 10 females) with a mean age of 22.45 ± 0.92 years. The control group, on the other hand, consisted of 20 subjects (6 males, 14 females) with a mean age of 22.85 ± 0.98 years. The mean value of occlusal canting in the treatment group was 3.02 ± 0.17 mm whereas that in the control group was 4.15 ± 0.34 mm. The mean treatment duration in the treatment group was 15.9 ± 5.48 months whereas that in the control group was 32.9 ± 14.05 months

As shown in Table 5, in the treatment group (those who were subjected to the surgery-first approach), statistically significant changes were observed for all the measurements when the values obtained before surgery (T0) and immediately after surgery (T1) were compared. Statistically significant changes were also observed for U1-SRP, R body height, and L body height when the values obtained immediately after surgery (T1) and 6 months after surgery (T2) were compared. Statistically significant changes were not observed for the other measurements.

**Table 5:** Mean and standard deviation of linear and angular measurement in the experimental group

Measurements	T0–T1			T1–T2		
	Mean	SD	*P* value	Mean	SD	*P* value
A-SRP	− 0.248	1.677	0.010*	− 0.050	1.487	0.884
**U1-SRP**	− **0.384**	**1.572**	0.002*	**0.566**	**1.313**	0.016*
Me-SRP	1.202	2.436	0.000*	0.859	2.907	0.214
Pog-CP	6.029	5.988	0.000*	− 0.814	3.005	0.253
**R body height**	**0.257**	**1.093**	0.001*	**1.050**	**1.481**	0.006*
**L body height**	**1.348**	**1.828**	0.005*	**0.972**	**1.636**	0.018*
R maxillary height	3.941	1.995	0.000*	0.321	1.391	0.327
L maxillary height	2.525	2.009	0.000*	0.548	1.876	0.219
R ramus length	1.536	1.783	0.000*	1.372	4.782	0.227
L ramus length	− 0.020	3.097	0.000*	1.498	3.811	0.104
R frontal ramal inclination	− 1.094	3.694	0.000*	− 0.114	2.663	0.854
L frontal ramal inclination	− 4.005	2.596	0.000*	− 0.021	2.491	0.970

Statistically significant changes were also observed for U1-SRP, R body height, and L body height when the values obtained immediately after surgery (T1) and 6 months after surgery (T2) were compared. Statistically significant changes were not observed for the other measurements (*P < 0.05)

As shown in Table 6, on the other hand, in the control group (those who were not subjected to the surgery-first approach), statistically significant changes were observed for all the measurements when the values obtained before surgery (T0) and immediately after surgery (T1) were compared. Statistically significant changes were also observed for R body height and L body height when the values obtained immediately after surgery (T1) and 6 months after surgery (T2) were compared. Statistically significant changes were not observed for the other measurements.

**Table 6:** Mean and standard deviation of linear and angular measurement in the control group

Measurements	T0–T1			T1–T2		
	Mean	SD	*P* value	Mean	SD	*P* value
A-SRP	− 0.165	1.554	0.020*	0.062	1.697	0.512
U1-SRP	− 0.261	1.128	0.003*	0.371	1.158	0.075
Me-SRP	0.947	1.173	0.005*	0.695	1.382	0.158
Pog-CP	4.273	3.286	0.000*	− 0.597	2.038	0.106
**R body height**	**0.424**	**1.824**	0.000*	**1.139**	**1.021**	0.019*
**L body height**	**1.205**	**1.828**	0.005*	**0.604**	**0.947**	0.023*
R maxillary height	2.373	1.886	0.000*	0.583	0.816	0.962
L maxillary height	3.163	2.353	0.000*	0.729	1.238	0.219
R ramus length	2.274	3.116	0.000*	1.409	2.537	0.448
L ramus length	0.162	2.293	0.000*	1.943	3.093	0.356
R frontal ramal inclination	− 2.312	2.301	0.000*	− 0.568	1.193	0.279
L frontal ramal inclination	− 3.061	3.743	0.000*	− 0.081	1.943	0.628

Statistically significant changes were also observed for R body height and L body height when the values obtained immediately after surgery (T1) and 6 months after surgery (T2) were compared. Statistically significant changes were not observed for the other measurements (*P < 0.05)

As shown in Table 7, a significant difference was found between the control and treatment groups with respect to U1-SRP, in terms of the postsurgical change (T1–T2).

**Table 7:** Comparison of postsurgical change (T1–T2) in experimental and control groups

Measurements	Experimental group		Control group		*P* value
	Mean	SD	Mean	SD	
A-SRP	− 0.050	1.487	0.062	1.697	0.195
**U1-SRP**	**0.566**	**1.313**	**0.371**	**1.158**	0.001*
Me-SRP	0.859	2.907	0.695	1.382	0.505
Pog-CP	− 0.814	3.005	− 0.597	2.038	0.813
R body height	1.050	1.481	1.139	1.021	0.132
L body height	0.972	1.636	0.604	0.947	0.263
R maxillary height	0.321	1.391	0.583	0.816	0.142
L maxillary height	0.548	1.876	0.729	1.238	0.780
R ramus length	1.372	4.782	1.409	2.537	0.216
L ramus length	1.498	3.811	1.943	3.093	0.966
R frontal ramal inclination	− 0.114	2.663	− 0.568	1.193	0.203
L frontal ramal inclination	− 0.021	2.491	− 0.081	1.943	0.423

Significant difference was found between the control and treatment groups with respect to U1-SRP, in terms of the postsurgical change (T1–T2) (*P < 0.05)

## Discussion

The form of facial asymmetry was traditionally analyzed using PA cephaloradiographs [22–25]. In such reports, the frequency of facial asymmetry among patients with facial deformities showed a 21–67% distribution. Severt and Proffit [22] reviewed the medical records of 1460 patients with maxillofacial deformities and reported that 34% of them showed facial asymmetry, with a high prevalence of facial asymmetry (40%) in patients with class III malocclusion. Tani et al. [24] analyzed the PA cephaloradiographs of 239 patients with maxillofacial deformities and reported that 28% of them also had facial asymmetry.

During the treatment of patients with facial asymmetry, preoperative orthodontic treatment is first provided to remove the dental compensation of the maxillary and mandibular dentition. The midline of the anterior teeth is aligned with the midline of the jaws, and the inclinations of the anterior teeth and the occlusal plane are matched. This increases the predictability of postoperative teeth movement and simplifies the surgical planning [26]. Also, by matching the left and right posterior torques and increasing the overjet of the non-involved canines, asymmetry worsening is prevented by occlusal interference during surgery [26]. Then, through orthognathic surgery, the midline of the jaw and that of the anterior are aligned. Complete decompensation may not be achieved, however, due to the function of occlusion, the direction of natural compensation, and the strength of the muscles. This can result in difficulties in orthognathic surgery and postoperative orthodontic treatment.

Treating patients with facial asymmetry poses more difficulties than treating those without. If the vertical difference between the first molars of the left and right maxilla with respect to the FH plane is not accurately assessed in preoperative orthodontic treatment, or if the height difference between the left and right mandibular occlusal planes with respect to the mandibular plane is not accurately assessed and only mandibular surgery is performed, the asymmetry cannot be completely corrected [27]. If the cross-decompensation of dentition in preoperative orthodontic treatment is incomplete, postoperative skeletal asymmetry will remain even after achieving satisfactory occlusion through surgery. Also, an asymmetric maxillary arch may appear in the posterior maxilla despite carefully performed preoperative orthodontic treatment; as a result, asymmetry may remain in the mandibular angle and ramus mandibulae due to an inappropriately positioned mandible [28].

Several studies have been reported on the overall trend of relapse after facial asymmetry surgery. In 1997, Severt and Proffit [22] reported a 40% regression rate after orthognathic surgery. In 2002, Lai et al. [29] reported that menton lateral relapse occurred in up to 24% of the cases. It was reported that the cause of this was the difference between the amount of mandibular retraction in the left and right sides and the displacement of the mandibular condyle. In 2009, however, Ko et al. [30] reported symmetric results and skeletal stability of the chin after orthognathic surgery.

Many studies on the surgery-first approach have been reported of late. Compared with the traditionally used method, the surgery-first approach has many advantages, such as increased patient cooperation, efficient decompensation, and decreased treatment duration [31]. In 2011, Liou et al. [32] presented a guideline for model surgery and orthodontic treatment during the implementation of the surgery-first approach [33]. In 2011, Hwang et al. [34] reported that horizontal and vertical skeletal stability can be achieved after orthognathic surgery through the surgery-first approach in patients with class III skeletal malocclusion. Thus, most of the published papers are on the class III skeletal malocclusion [15–18, 31, 33, 34], and there is no paper as yet on asymmetry.

In this study, statistically significant changes were observed in U1-SRP, R body height, and L body height during the T1–T2 period when orthognathic surgery through the surgery-first approach was performed, and statistically significant changes were observed in R body height and L body height during the T1–T2 period when the traditional method was used. These results suggest that orthognathic surgery through the surgery-first approach provides a degree of skeletal stability comparable to that provided by the traditional method and that both the treatment and control groups in this study retained skeletal stability postoperatively for 6 months. In this study, the mean duration of the treatment period for orthognathic surgery through the surgery-first approach was 15.9 months whereas the mean treatment period for the cases without the surgery-first approach was 32.9 months. Thus, the surgery-first approach cases showed a shorter treatment period than the traditional method cases. In 2011, Hwang et al. reported that in class III malocclusion patients, horizontal and vertical bone stability was obtained after orthognathic surgery through the surgery-first approach [34].

In the case of patients with asymmetry, surgery is performed by setting the anterior midline to predict the change in the dental axis of the anterior and posterior maxilla and mandible owing to the postoperative compensation, and orthodontic treatment is provided after surgery. In those cases where orthognathic surgery was performed using the surgery-first approach, statistically significant differences were observed in U1-SRP during the T1–T2 period. Such results may be due to the crowding of the anterior teeth, the dental spacing, and the deviation of the dental axis from the basal bone. When performing preoperative orthodontic treatment, the orthodontist will decompensate the teeth to the basal bone. For these reasons, it is important to set up the occlusion and the setting of the maxillary incisors in the preoperative planning through the surgery-first approach. The posterior teeth axis is also one of the factors that make it difficult to set the occlusion in the surgery-first approach.

The subjects of this study were limited to patients who did not have severe preoperative lateral posterior dental compensation and those who did not have posterior cross-bite. Also, a stent was worn for up to 10 weeks to resolve the unstable occlusion in the posterior after surgery, and when necessary, the surgical stent was fixed at the anterior maxilla after cutting out the posterior section of the first premolars, to address the transverse disharmony in the posterior maxilla and mandible using the cross elastics of the posterior. As mentioned earlier, clinical difficulties were observed in postoperative orthodontic treatment, but it is believed that these were not statistically significant.

## Conclusion

In the patients with asymmetry in this study, orthognathic surgery through the surgery-first approach showed similar results in terms of postoperative skeletal stability as the traditional method that is used after orthodontic treatment, but a significant lateral variation of the upper midline was observed. In addition, a shorter average treatment duration was observed. To stabilize the unstable occlusion after surgery, increased wearing of the stent and proactive rubber guidance will be needed.

## Data Availability

All data generated or analyzed during this study are included in this published article [and its supplementary information files].

## Abbreviations

3D: 3-Dimensional
2D: 2-Dimensional
RAP: Regional Acceleratory Phenomenon
DCT: Dental cone-beam tomography
MMF: Maxillomandibular fixation
